# Synthetic GM1 improves motor and memory dysfunctions in mice with monoallelic or biallelic disruption of GM3 synthase

**DOI:** 10.1002/2211-5463.13669

**Published:** 2023-07-13

**Authors:** Suman Chowdhury, Ranjeet Kumar, Evelyn Zepeda, Shawn DeFrees, Robert Ledeen

**Affiliations:** ^1^ Department of Pharmacology, Physiology, and Neuroscience Rutgers, The State University of New Jersey Newark NJ USA; ^2^ Seneb BioSciences, Inc. Wilton CT USA

**Keywords:** GM1 ganglioside, GM3 synthase, memory impairment, motor impairment, Parkinson's disease

## Abstract

This study attempts to answer the question of whether mice with biallelic and monoallelic disruption of the *St3gal5* (GM3 synthase) gene might benefit from GM1 replacement therapy. The GM3 produced by this sialyltransferase gives rise to downstream GD3 and the ganglio‐series of gangliosides. The latter includes the a‐series (GM1 + GD1a), which has proved most essential for neuron survival and function (especially GM1, for which GD1a provides a reserve pool). These biallelic mice serve as a model for children with this relatively rare autosomal recessive condition (*ST3GAL5*−/−) who suffer rapid neurological decline including motor loss, intellectual disability, visual and hearing loss, failure to thrive, and other severe conditions leading to an early death by 2–5 years of age without supportive care. Here, we studied both these mice, which serve as a model for the parents and close relatives of these children who are likely to suffer long‐term disabilities due to partial deficiency of GM1, including Parkinson's disease (PD). We find that the movement and memory disorders manifested by both types of mice can be resolved with GM1 application. This suggests the potential therapeutic value of GM1 for disorders stemming from GM1 deficiency, including GM3 synthase deficiency and PD. It was noteworthy that the GM1 employed in these studies was synthetic rather than animal brain‐derived, reaffirming the therapeutic efficacy of the former.

## Graphical abstract

GM3 synthase deficiency is caused by biallelic disruption of *St3gal5* gene. It is rare and characterized by intellectual disability, early‐onset choreoathetosis, hearing and visual impairments, and failure to thrive. In this study, GM3 synthase‐deficient mice were shown to have movement disorders and short‐term spatial memory defects. GM1 alone improved movement and memory dysfunctions despite deletion of all ganglio‐series gangliosides.

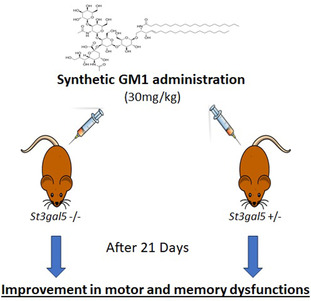

**Abbreviations**
BBB: blood–brain barrier
BSS: basic salt solution
CNS: central nervous system
GM1‐OS: oligosaccharide GM1
HT: heterozygous
IP: intraperitoneal
KO: knockout
PD: Parkinson's disease
PNS: peripheral nervous system
SEM: standard error of the mean
WT: wild‐type

Gangliosides are the subgroup of glycosphingolipids whose main structural feature is the presence of one or more sialic acids attached to the oligosaccharide chain [1]. They appear to be ubiquitous in all vertebrate cells but especially abundant in neurons, rendering the brain the heaviest repository [2]. There is substantial evidence of their essentiality for neuronal function and viability, as is well‐illustrated in those neurological disorders characterized by the absence of one or more ganglioside groupings, for example, absence of the ganglio‐series gangliosides in early‐onset hereditary spastic paraplegia due to disruption of the *B4GALNT1* (GM2/GD2 synthase) gene with loss of the ganglio‐series gangliosides (principally GM1, GD1a, GD1b, GT1b as well as the c‐series) [3]. Mice with this lesion are somewhat less affected than humans but were shown to manifest multiple symptoms of Parkinson's disease (PD) [4]. An even more serious condition in humans arises from disruption of the *ST3GAL5* (GM3 synthase) gene which results in deletion of gangliosides GM3 and GD3 along with the ganglio‐series [5, 6, 7] while mice with this lesion also suffer more debilitation than the above mice [8, 9].

The homozygous GM3 synthase‐deficient mice are one subject of this study, with focus on movement disorders and defective short‐term spatial memory, which are also present in *B4galnt1*(−/−) and *B4galnt1*(+/−) mice [4, 10]. The question we are addressing is whether GM1 alone, despite deletion of all ganglio‐series gangliosides, is capable of correcting the Parkinsonian‐like movement disabilities and defective short‐term memory, as it was in the latter two conditions [4, 10, 11]. Loss of GM3 synthase results from biallelic disruption of the *ST3GAL5* gene, which encodes a sialyltransferase that produces GM3 [6, 7, 12]. The latter in turn gives rise to the ganglios‐series of gangliosides. The principal features in children with this relatively rare autosomal recessive condition include intellectual disability, early‐onset choreoathetosis, visual and hearing impairments, failure to thrive, and cutaneous dyspigmentation [5, 13]. It occurs most prevalently in Old Order Amish communities [14] but in non‐Amish patients as well [15]. Gene modification is an obvious therapeutic approach [16] but ganglioside replacement therapy in which patients are administered one or more of the missing gangliosides deemed most essential for neuronal function is also worthy of consideration. In that regard, ganglioside GM1 is receiving primary attention owing to the several functions it mediates in preservation of neuronal function and viability [17, 18].

Less attention has been given to individuals or mice with monoallelic disruption of the *ST3GAL*5 gene (*St3gal5*+/−) although they too are likely suffering neurological disabilities due to partial deletion of the same gangliosides as for the homozygous knockout (KO) mice with complete deletion. We show in the present study that is indeed the case for *St3gal5+/−* heterozygous (HT) mice whose movement disorder and short‐term spatial memory loss are also corrected by GM1 administration.

## Methods

### Preparation of synthetic GM1

Synthetic GM1 was provided by Seneb BioSciences, Inc., which prepared it via metabolically engineered *Escherichia coli* as described previously [19, 20]. A key characteristic of synthetic GM1 is the identity of its oligosaccharide to brain GM1 oligosaccharide, while the ceramide structure can vary in certain ways (see Discussion). See international patent WO: 2006/03225 A2 for details on the synthesis procedure.

### Mice and genotyping

A breeding pair of *St3gal5+/−* was a gift from R. Yu. Mice were bred and maintained in the Rutgers University, New Jersey Medical Science Animal Facility with 12 h light/dark cycles. Mice genotyping was done by Transnetyx Inc. (Cordova, TN, USA) using tail snips. Biallelic (*St3gal5*−/−) KO male mice at 7–10 months of age (*n* = 5) along with wild‐type (WT) of same age (*n* = 8) and monoallelic (*St3gal5*+/−) HT male mice at 10–12 months of age (*n* = 11) along with WT of same age (*n* = 6) were used for the study (female mice were found to be unreliable for the study). The limited number of KO mice for the study, as mentioned, is because these mice suffered low survival rates due to their multiple pathologies and dysfunctions. KO and HT mice were administered synthetic GM1 at a dose of 30 mg·kg^−1^ daily via intraperitoneal (IP) injection over a period of 21 days; basic salt solution (BSS) was administered similarly to WT mice. All animal studies were conducted under a protocol (PROTO201800212) approved by the Rutgers University Institutional Animal Care and Use Committee (IACUC) in accordance with the guidelines of the Rutgers Institutional Animal Care and Use Committees.

### Motor and memory impairment tests

#### Grip duration test

To determine the extent of motor impairment, the mouse was allowed to grasp a narrow horizontal rod located 50 cm above a pillow with its forepaws. This procedure was carried out in order to measure the amount of time required to fall from the rod [4, 21]. A gentle hold on the tail of the mice was used to prevent them from climbing on their hind legs. The test was performed three times successively with a 30‐min rest interval between each trial. Based on the results, an average duration was calculated.

#### Beam traversal test

Beam traversal test was used to test motor coordination and balance. A wooden beam (width 12 mm, length 90 cm) was placed horizontally. The time spent traversing the beam and the number of foot slips was measured [22, 23]. Mice were trained three times before the first trial, followed by five consecutive trials.

#### T maze test

An examination of short‐term spatial memory capacity in mice was conducted using a T maze (similar to Y maze) forced‐trial spontaneous alternation test [24]. The mouse was placed into the center arm and forced into one arm by blocking another arm with a barrier. Once the mouse enters the open arm, it is held there for 45 s. The mouse is then removed and placed into a stimulus‐free cage for a 60 s retention interval and again placed back into the center arm. A normal mouse's instinct is to choose the new arm for exploration, since it is expected to retain the memory. The exploration of the initial arm was recorded as incorrect, and the exploration of the novel arm was recorded as correct. Each mouse was subjected to three trials, separated by an interval of 1 h. The score was calculated as a percentage of spontaneous alternation rate.

### Statistical analysis

All the data are expressed as mean ± SEM (standard error of the mean). Statistical analysis was performed by one‐way ANOVA followed by Bonferroni's multiple comparisons tests. *P*‐value < 0.05 was considered significant.

## Results

**Fig. 1:**
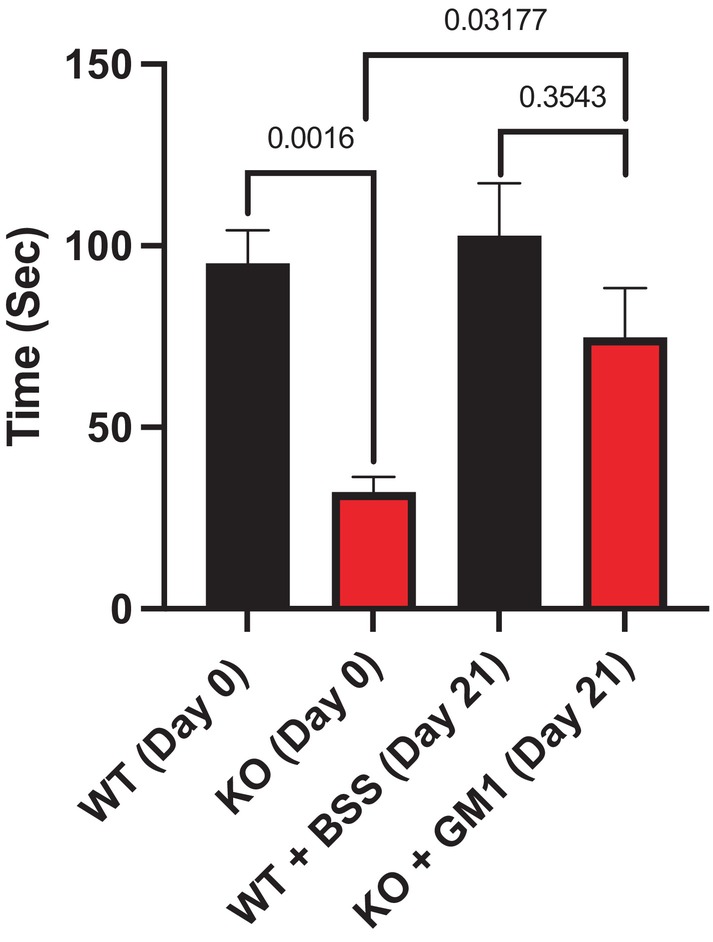
Improvement of grip duration test by synthetic GM1 in KO mice. KO mice at 7–10 months of age (*n* = 5) were IP injected with 30 mg·kg^−1^ GM1 and WT mice of the same age (*n* = 8) were IP injected with BSS daily for 21 days. *P*‐value < 0.05 is considered significant. The test was done at D 0 to check the basal state and at D 21 to check the effect of GM1 at the end of treatment. All data are presented as mean ± SEM and were evaluated by one‐way ANOVA followed by Bonferroni's multiple comparisons tests.

**Fig. 2:**
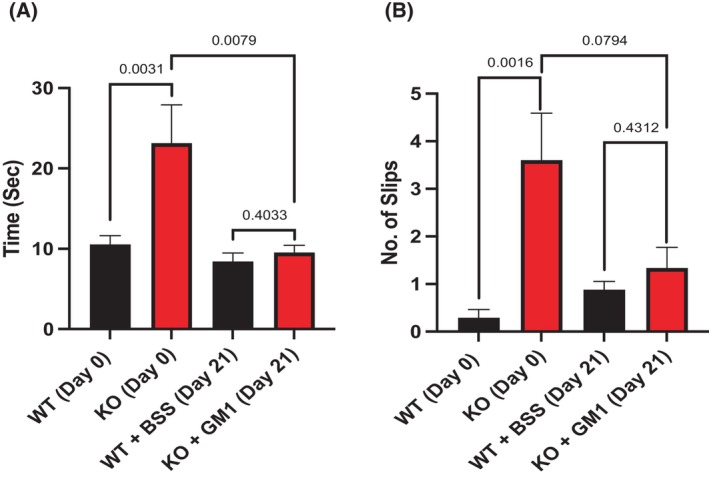
Improvement of beam traversal test by synthetic GM1 in KO mice. KO mice at 7–10 months of age (*n* = 5) were IP injected with 30 mg·kg^−1^ GM1 and WT mice of the same age (*n* = 8) were IP injected with BSS daily for 21 days. (A) Time taken by mice to cross the beam, and (B) number of times mice slip while crossing the beam. *P*‐value < 0.05 is considered significant. The test was done at D 0 to check the basal state and at D 21 to check the effect of GM1 at the end of treatment. All data are presented as mean ± SEM and were evaluated by one‐way ANOVA followed by Bonferroni's multiple comparisons tests.

**Fig. 3:**
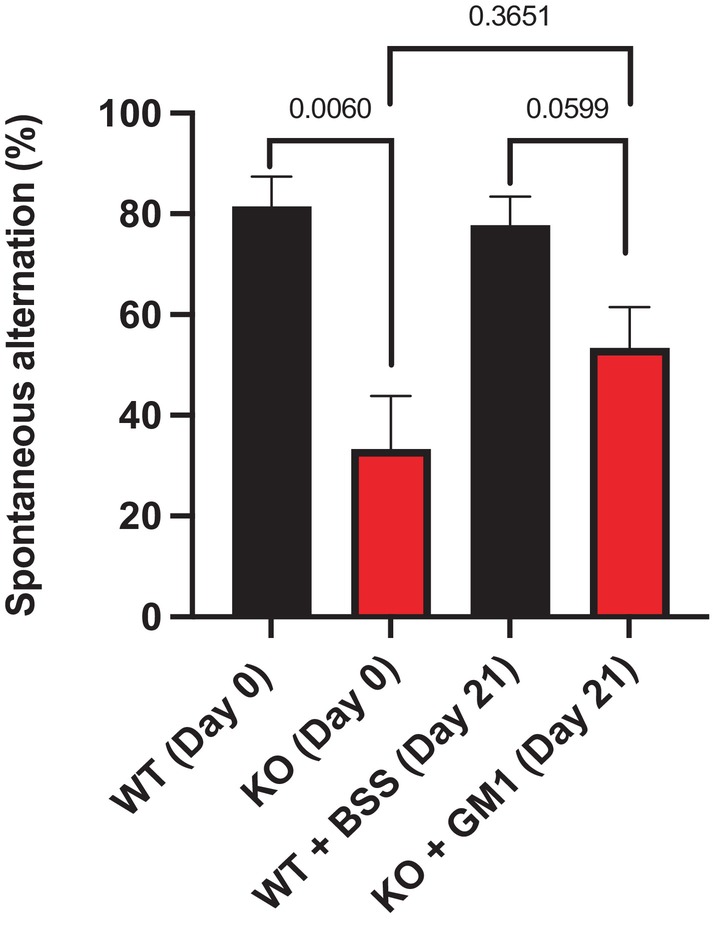
Improvement of short‐term spatial memory impairment (T maze test) by synthetic GM1 in KO mice. KO mice at 7–10 months of age (*n* = 5) were IP injected with 30 mg·kg^−1^ GM1 and WT mice of the same age (*n* = 8) were IP injected with BSS daily for 21 days. *P*‐value < 0.05 is considered significant. The test was done at D 0 to check the basal state and at D 21 to check the effect of GM1 at the end of treatment. All data are presented as mean ± SEM and were evaluated by one‐way ANOVA followed by Bonferroni's multiple comparisons tests.

Homozygous GM3 synthase‐deficient (KO) mice showed significantly poorer performance than WT mice on the grip duration (Fig. 1) and beam traversal tests (Fig. 2), as well as short‐term spatial memory test (Fig. 3). The tests were performed at D 0 to check the basal state and at D 21 to check the effect of GM1 at the end of treatment. The differences in performance largely disappeared following GM1 treatment, during which the KO mice improved significantly. This was true despite the limited number of KO mice for the study; as mentioned, these mice suffered low survival rates due to their multiple pathologies and dysfunctions. Importantly, GM1 treatment improved KO mice performance in grip duration test (*P* = 0.032) and beam traversal test significantly (*P* = 0.0079); the GM1 treatment elevated the T maze performance to largely eliminate the significant difference compared to WT.

**Fig. 4:**
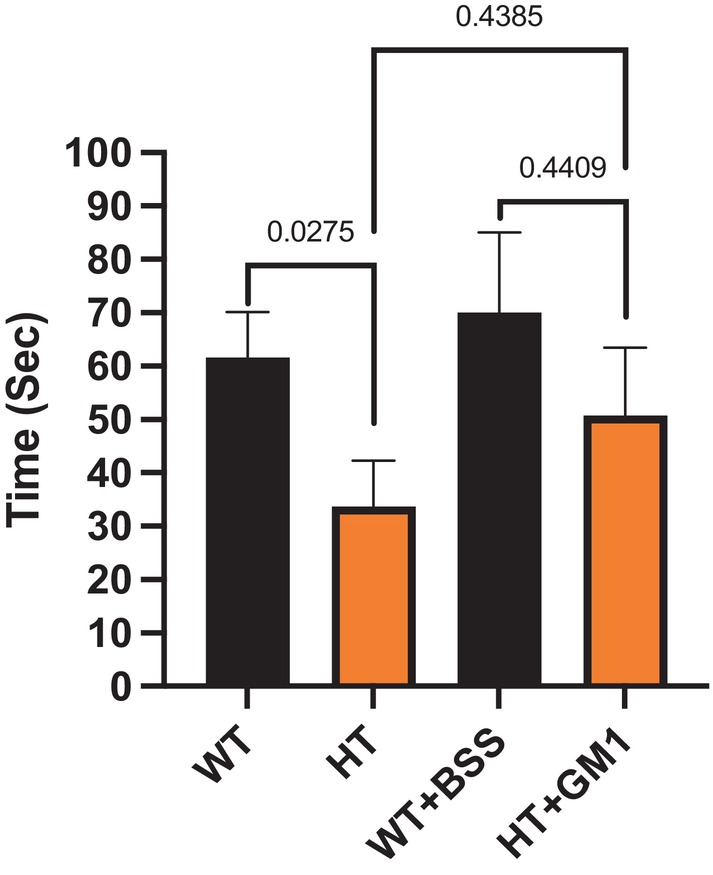
Improvement of grip duration test by synthetic GM1 in HT. HT mice at 11–13 months of age (*n* = 11) were IP injected with 30 mg·kg^−1^ GM1 and WT mice of the same age (*n* = 6) were IP injected with BSS daily for 21 days. *P*‐value < 0.05 is considered significant. The test was done at D 0 to check the basal state and at D 21 to check the effect of GM1 at the end of treatment. All data are presented as mean ± SEM and were evaluated by one‐way ANOVA followed by Bonferroni's multiple comparisons tests.

**Fig. 5:**
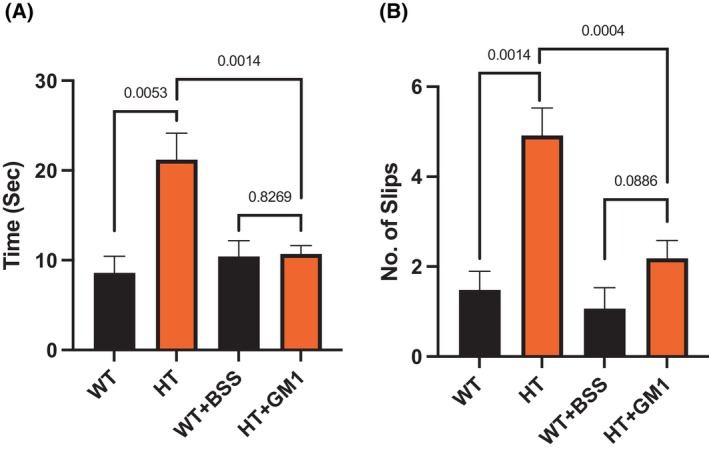
Improvement of beam traversal test by synthetic GM1 in HT mice. HT mice 11–13 months of age (*n* = 11) were IP injected with 30 mg·kg^−1^ GM1 and WT mice of the same age (*n* = 6) were IP injected with BSS daily for 21 days. (A) Time taken by mice to cross the beam, and (B) number of times mice slip while crossing the beam. *P*‐value < 0.05 is considered significant. The test was done at D 0 to check the basal state and at D 21 to check the effect of GM1 at the end of treatment. All data are presented as mean ± SEM and were evaluated by one‐way ANOVA followed by Bonferroni's multiple comparisons tests.

**Fig. 6:**
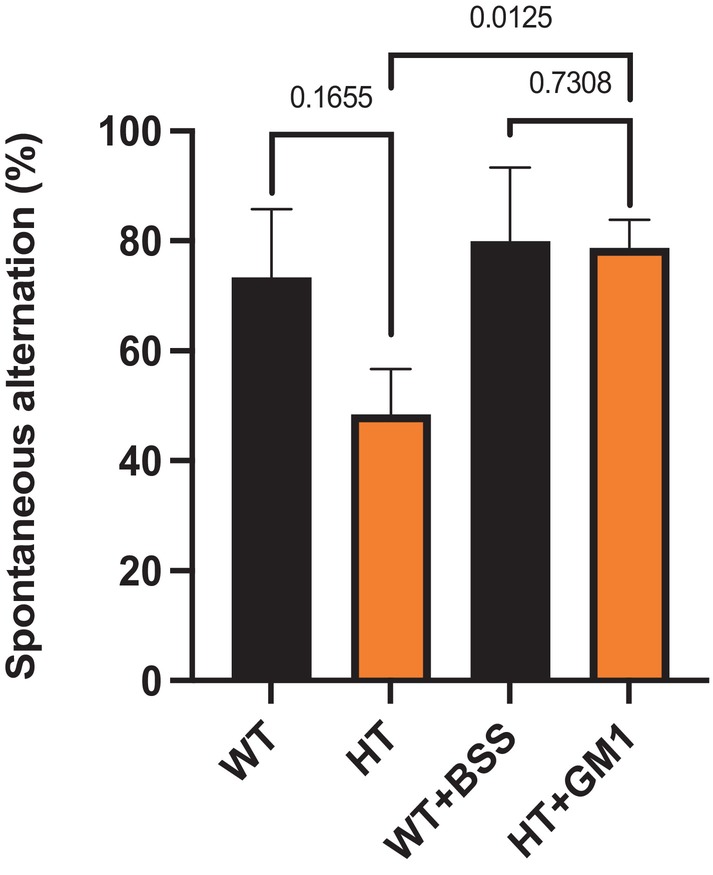
Improvement of short‐term spatial memory impairment (T maze test) by synthetic GM1 in HT mice. HT mice 11–13 months of age (*n* = 11) were IP injected with 30 mg·kg^−1^ GM1 and WT mice of the same age (*n* = 6) were IP injected with BSS daily for 21 days. *P*‐value < 0.05 is considered significant. The test was done at D 0 to check the basal state and at D 21 to check the effect of GM1 at the end of treatment. All data are presented as mean ± SEM and were evaluated by one‐way ANOVA followed by Bonferroni's multiple comparisons tests.

The heterozygous GM3 synthase‐deficient mice (HT) suffered fewer pathologies and dysfunctions than the KO mice but still showed significant impairments prior to GM1 treatment in the three tests employed (Figs 4, 5, 6). In the grip duration test, the HT mice showed significant impairment prior to GM1 treatment (*P* = 0.0275) and this impairment largely disappeared with GM1 treatment (*P* = 0.4409). Although GM1 appeared to improve performance, the change was not significant (*P* = 0.4385).

Beam traversal was significantly impaired prior to GM1 treatment (*P* = 0.0053), but this impairment disappeared with GM1 treatment (Fig. 5). In this case, GM1‐induced change was highly significant (*P* = 0.0014). The foot slippage test was significantly impaired (more slippage, *P* = 0.0014) prior to GM1 treatment, which largely disappeared after GM1 treatment (*P* = 0.0004).

The results for the T maze test were slightly different, in that HT mice did not show significant difference compared with WT mice prior to GM1 treatment (*P* = 0.1655). However, the GM1 treatment did induce significant improvement in this test (*P* = 0.0125).

## Discussion

The results showed significant difference between KO and WT mice in the grip duration test prior to treatment and no significant difference following GM1 treatment for 21 days. The same was observed for beam traversal, the other movement disorder tested; the latter included both traversal time and the number of slips during the walk. These observed changes were significant despite the limited number of KO mice available at this testing time (the KO homozygous mice suffer high mortality rates at relatively young ages). Despite the severity of disease in the KO mice at the age of testing, GM1 treatment was able to correct the motor and cognitive deficits. These results suggest that even humans with late‐stage GM3 synthase deficiency might benefit from GM1 replacement therapy. Similar results were obtained with the T maze, short‐term spatial memory test, despite similar limitation in KO mouse number.

Thus, GM1 replacement therapy may receive consideration as an alternative to gene therapy (which has its own disadvantages), although more work is required to test the ability of GM1 to resolve other possible dysfunctions in the KO mice (e.g., visual and hearing impairments). It may be noted that while GM1 and GD1a together comprise 80% or more of total brain gangliosides [12], the principal function of GD1a appears to be that of reserve pool for GM1 since it is converted to the latter via Neu3 neuraminidase which has membrane locus close to that of those two gangliosides [25].

It was of interest that heterozygous mice (HT), *St3gal5*(+/−) were also responsive to GM1 treatment. In the case of short‐term spatial memory, although there was an observed difference between HT and WT, this did not reach significance prior to treatment. However, GM1 treatment did cause virtual elimination of the difference between HT and WT and effected significant improvement compared with untreated HT (Fig. 5). The modest difference prior to treatment was likely due to partial depletion of GM1 attributable to monoallelic disruption of this gene, similar to that occurring in *B4galnt1*(+/−) mice [4, 10]. This indicates that both parents and possibly other close relatives of the afflicted children would be at risk of expressing subnormal GM1 with resulting health consequences. Primary among the latter is PD shown to be characterized by neuronal pathologies due to subnormal GM1 in both the central nervous system (CNS) and peripheral nervous system (PNS) [26, 27] as well as non‐neuronal cells [28]. This in turn correlates with the fact that Old Order Amish communities, a major repository of persons with disrupted *ST3GAL5* [14] also manifest the highest PD incidence in the world [29]. This suggests the potential advantage of monitoring those parents and close relatives for early detection of PD (prodromal stage) with corresponding therapeutic advantages.

In KO mice, GM1 was shown to have a greater effect than in HT mice possibly due to the KO mouse blood–brain barrier (BBB) being more damaged, and therefore more permeable to GM1, whereas BBB of HT mice is likely more intact, limiting GM1's central passage. GM1 has been shown to be inefficient at crossing the BBB [30], and its daily IP administration did not provide statistically significant improvement in PD symptoms [10]. Therefore, in HT mice GM1 was possibly not able to exert its full neuroprotective effects due to the low quantity of GM1 that crossed the BBB.

It is worth noting that the GM1 employed in this study was synthetic (see Methods) rather than animal‐derived to avoid possible prion contamination. The oligosaccharide of synthetic GM1 is identical to that of animal‐derived GM1. Similar synthetic GM1 was also employed in an earlier study [21], which showed the synthetic form to be as effective as the brain‐derived form in resolving both CNS and PNS localized pathologies in an animal model of PD. This was despite the fact that the ceramide in synthetic GM1 contains a single form of sphingosine (d18:1) in contrast to brain‐derived GM1 which contains that in addition to an approximately equivalent amount of C20‐sphingosine (d20:1) [31]. In addition, a single fatty acid (18:0) is present in the synthetic GM1 in contrast to the mixture of fatty acids (low amounts of 18:1, 16:0, and 20:0 in addition to the predominant 18:0) [2] in animal brain GM1. Interestingly, ceramide from PD‐involved portions of PD patient brains contained significant amounts of shorter‐chain fatty acids [32], though the pathophysiological significance of this difference is not yet known. In any case, the oligosaccharide portion of GM1 devoid of ceramide (GM1‐OS) was shown as effective as GM1 itself in therapy for an animal model of PD [33]. That is in addition to a variety of *in vitro* neuronal functions mediated by GM1‐OS [34]. Since it (GM1‐OS) crosses the BBB more readily than GM1 [35], this suggests GM1‐OS is worthy of consideration. The oligosaccharide portion of synthetic GM1 is identical to animal‐derived GM1 [34]. Thus, there is substantial justification for the use of synthetic GM1 for therapeutic purposes in such studies.

## Conclusion

The present study focuses on mice with monoallelic or biallelic disruption of the *St3gal5* (GM3 synthase) gene. Both mice suffer neurological dysfunctions due to the total or partial deletion of GM3, GM1, and other gangliosides. The KO mice were severely affected by the time were treated with GM1. Our findings suggest that GM1 treatment in both KO and HT mice, despite the severity of the disease in the KO mice, can alleviate motor and short‐term spatial memory dysfunctions and may lead to the development of a clinically relevant treatment for GM3 synthase deficiency. Our results of treatment with GM1 would be equivalent to treating end‐stage human disease.

## Conflict of interest

The authors declare no conflict of interest.

### Peer review

The peer review history for this article is available at https://www.webofscience.com/api/gateway/wos/peer‐review/10.1002/2211‐5463.13669.

## Author contributions

SC and RL conceived and designed the project. SC, RK, and EZ acquired the data. SC, RK, SD, and RL analyzed and interpreted the data. SC and RL wrote the paper. SC, RK, SD, and RL proofread the paper.

## Data Availability

The data that support the findings of this study are available from the corresponding author (ledeenro@njms.rutgers.edu) upon reasonable request.
